# Peer Review of “The Role of Anxiety and Prosocial Behaviors on Adherence Behaviors to Prevent COVID-19 in University Students in the United States: Cross-Sectional Study”

**DOI:** 10.2196/59430

**Published:** 2024-05-27

**Authors:** Femi Qudus Arogundade, Syeda Azra, Myron Pulier, Limegreen Ram, Daniela Saderi, Magdalena Tomaskova

**Affiliations:** 1 Public Health U, Abuja, Nigeria; 2 SKR Government College, Yogi Vemana University, Kadapa, India; 3 Rutgers New Jersey Medical School, Newark, NJ, United States; 4 Division of Psychiatry, University College London, London, United Kingdom

**Keywords:** prosocial behavior, COVID-19, anxiety, COVID-19 prevention, preventive health behavior, adherence to prevention

*This is the peer-review report for “The Role of Anxiety and Prosocial Behaviors on Adherence Behaviors to Prevent COVID-19 in University Students in the United States: Cross-Sectional Study.”*

## Round 1 Review

This review is the result of a virtual collaborative live review discussion organized and hosted by PREreview and JMIR Publications. The discussion was joined by 16 people: 1 author, 2 facilitators, 2 members of the JMIR Publications team, and 11 live review participants. Aishah Ibrahim and Ananya Ananthakrishnan wished to be recognized for their participation in the live review discussion, even though they have not contributed to authoring the review below. We thank all participants who contributed to the discussion and made it possible for us to provide feedback on this preprint.

### Summary

The study [1] investigates the complex interplay between anxiety (both state and trait), prosocial behaviors, and adherence to COVID-19 preventive measures among college students. While overall prosocial behaviors did not directly correlate with anxiety, a seemingly significant crossover effect emerged in relation to public prosocial behaviors, suggesting that individuals with lower self-oriented tendencies exhibited increased adherence behaviors under heightened state anxiety. The study used a quantitative research design with a sample of 54 undergraduate students, using online questionnaires to measure various psychological factors and preventive behaviors. Individuals with high anxiety showed increased adherence to preventive measures, contrary to the hypothesized moderating effect of prosocial behaviors. Overall, the reviewers appreciated the effort and recognized the challenges of conducting a research study in the context of an unprecedented social condition. However, the findings are challenged by weak effect sizes, multiple comparisons, and unclear appropriateness of using prosocial behaviors as a moderating variable. The study underscores the psychological impact of the COVID-19 pandemic on college students and suggests the need for further exploration into the nuanced relationships between anxiety, prosocial behaviors, and adherence to public health guidelines. Despite its strengths in data collection and questionnaire use, limitations such as a narrow participant pool and reliance on self-reporting warrant cautious interpretation of the results. The study encourages future research to delve deeper into these intricate connections, offering insights into potential interventions for promoting adherence to COVID-19 preventive measures and beyond.

Below we list major and minor concerns that were discussed by participants of the live review, and where possible, we provide suggestions on how to address those issues.

### List of Major Concerns and Feedback

#### Small Sample Size and Mediation Analysis

One of the main concerns raised in the discussion was the small number of study participants. This is acknowledged as a limitation factor in the discussion, but what is less clear is if a mediation analysis is the right approach to analyze these data. One reviewer suggested the use of a multivariate analysis instead as it would take all variables into account without forcing potentially artificially generated causal mediations between variables that don’t show an obvious causal dependency. Another reviewer, however, felt that while this approach may work and it would be useful to explore, using a multivariate analysis may lead to overfitting in most covariates given the small sample size and given that 86% of subjects reported anxiety. Overall, the suggestion is for the authors to provide a rationale for selecting anxiety as a mediator variable over prosocial tendencies, or *vice versa*, and possibly explore other analyses and comment on the limitations of the approaches.

#### Uniform/Convenience Sampling

The reviewers acknowledged the study’s challenging circumstances and the effort to capture unique data. However, they expressed concern about generalizability, noting that all participants were undergraduates from one college. The demographic homogeneity of this group may limit applicability to diverse populations, raising caution about extrapolating results across age groups, educational backgrounds, and cultural contexts. The convenience sampling method may have introduced bias, as easily accessible participants might not represent the broader target population.

#### University Policy and Mask Mandates

The study doesn’t explicitly consider the potential impact of university campus policies during the pandemic, such as mask mandates and social distancing, on adherence behaviors. The findings may be influenced by the specific characteristics of the chosen university, including its restriction policies. For example, students on campus may wear face masks due to safety requirements when entering campus common spaces versus through their own personal decision process. Therefore, exercising caution in generalizing conclusions to broader contexts is suggested. Reviewers highlight the importance of future research with more diverse samples to enhance external validity, reinforcing the study’s overall robustness. This provides a constructive pathway for refining the study’s scope and applicability.

The study’s cross-sectional design and reliance on self-reporting introduce potential limitations in establishing causal relationships and accurate data collection. (In general, no retrospective exploratory study can show causality, asserting a causal relationship amounts to the post hoc ergo propter hoc logical fallacy.) The one-time nature of the study also limits insights into the dynamic nature of psychological factors and preventive behaviors over time. Caution should be exercised in interpreting the results, as correlation does not imply causation. Furthermore, reviewers don’t think that the results of this study can be used on their own to make any definitive public health policy recommendations.

#### Adherence Scores

The study mentions adherence to COVID-19 public health safety recommendations as an outcome variable. There is a need for more clarity on how the adherence scores were calculated, especially considering potential confounding factors such as university campus policies during the pandemic.

#### Missing Data

The study’s approach to handling missing data in nonmandatory survey questions is not explicitly discussed. This may impact the results and should be clarified.

#### Reliability Metrics

The study does not provide information on test-retest reliability, accuracy against a gold standard, or error of measurement for the Prosocial Tendencies Measure (PTM) reliability. Reliability induction from other studies is mentioned, but the study population’s specific reliability is not demonstrated. Without these critical reliability metrics, the study leaves a gap in the assessment of the psychometric properties of the PTM. Including such information would enhance the transparency and credibility of the study’s findings, allowing readers to better evaluate the reliability and validity of the instrument used to assess prosocial behaviors. Future research may consider providing a comprehensive assessment of the psychometric properties of measurement instruments to strengthen the methodological rigor and overall quality of the study.

#### Statistical Model and Data Selection

Some reviewers expressed concern related to the lack of transparency about how variables were selected as moderators or mediators, how some others (eg, age) were chosen to be excluded, and how others were chosen to be reported on from the cited “larger study.” Adding clarity around the rationale that led to making such choices would help the reader better contextualize the results.

Furthermore, a scoring guide for the CIS Survey would be helpful to add. There is a concern that a simple sum method may be biased because some questions may not be relevant to all subjects (eg, playdates only impact subjects that have childcare responsibilities).

#### Ethics

While the study mentions obtaining institutional review board approval and online passive consent, specific details regarding confidentiality, privacy safeguards, and participant understanding of risks are not thoroughly addressed.

Furthermore, it is not clear what the authors mean by “passive consent.” Ordinarily, the term involves a parent or guardian giving consent on behalf of someone deemed not competent to give consent (see Range L, Embry T, MacLeod T. Active and passive consent: a comparison of actual research with children. *Ethical Hum Sci Serv*. 2001 Spring;3(1):23‐31. PMID: 15278986). Were participants fully aware of what they were getting into, or does “passive” imply that consent was assumed by virtue of participants consenting to the terms of the “larger study”?

#### Data and Reproducibility

The study provides a moderate level of detail, but more specific information is needed for reproducibility. This includes additional demographic details, exact questionnaire wording, and more details on moderation analyses. The study would benefit from providing a more comprehensive set of demographic information about the participants such as age distribution, gender distribution, and other relevant characteristics. A richer demographic profile would contribute to a more nuanced understanding of the study population and facilitate comparisons with other research. Reviewers suggested adding available details to Table 1.

While the study mentions that data are available upon reasonable request, reviewers suggest considering providing additional information on how interested researchers can request the data, perhaps from the corresponding author or another designated contact. This could enhance transparency and facilitate potential collaborations or further scrutiny of the results.

### List of Minor Concerns and Feedback

#### Readability

Overall, the reviewers thought that the manuscript would benefit from a clearer explanation of key terms and recommended keeping the terminology consistent across the manuscript so as to help the reader better follow the narrative and interpret the findings. For example, there was some confusion among reviewers on the meaning of “public prosocial scale.”

#### Approach and Results

It may be helpful to show more information about some of the background variables. One question is if the deviation of age from a normal distribution is significant and thus a possible contributor to the study’s findings if age correlates with adherence or anxiety. Showing not only mean and SD but also median, quartiles, and range may provide a better feel for what the study population, or at least the participant sample, is like.

It may be useful to make explicit the assumptions underlying the modeling and parameters used for PROCESS, such as the degree of independence of the moderator.

#### Discussion

The authors may consider adding a section to the discussion to explore variables related to vaccine hesitancy and other factors (eg, sense of invincibility) as a suggestion for future research, expanding the scope beyond adherence to preventive measures.

Given the reliance on self-report measures, the reviewers suggest the authors discuss the potential impact of social desirability bias on participants’ responses. Addressing this concern would add transparency to the limitations of the study.

Reviewers suggest authors discuss how the results support following up further on correlations among PTM scales and on the possible moderator effect of public prosocial tendencies, with recommendations for including a broader set (explicitly listed) of potentially explanatory independent variables.

It may also be helpful to add some explanation of why the psychometric characteristics of the survey instruments as established in other studies can be trusted to be the same as used in this study (online, unsupervised, etc). Some reviewers found it concerning that this study found statistically significant pairwise associations between PTM subscales, and this should be addressed, perhaps with speculation about why this happened.

#### Figures and Tables

Consider using a 2 × 2 table in Figure 1 to illustrate the detected moderator effect.

#### Title

Given the concern about generalizability, a reviewer suggested the authors consider changing the title to “Adherence Behaviors to Prevent COVID: The Role of Anxiety and Prosocial Behaviors Amongst University Students in the US.”

### Concluding Remarks

We thank the authors of the preprint for posting their work openly and for graciously agreeing to have their work reviewed via this process. We also thank all participants of the Live Review call for their time and for engaging in the lively discussion that generated this review.

## Abbreviations

PTM: Prosocial Tendencies Measure
